# Docetaxel Micelles: A New Formulation to Diminish Hypersensitivity Reactions

**DOI:** 10.3390/pharmaceutics17020184

**Published:** 2025-02-02

**Authors:** Lanlan Xiang, Hao Wang, Jiajie Liu, Yuchen Shen, Yanfen Hu, Wenchen Che, Ran Li, Sisi Yang, Xin Teng

**Affiliations:** 1 National Material Experimental Teaching Demonstration Center, East China University of Science and Technology, 130 Meilong Road, Shanghai 200237, China; y82240247@mail.ecust.edu.cn (L.X.); y30230832@mail.ecust.edu.cn (H.W.); 20004084@mail.ecust.edu.cn (J.L.); 22010093@mail.ecust.edu.cn (Y.H.); 22010090@mail.ecust.edu.cn (W.C.); 22010050@mail.ecust.edu.cn (R.L.); 22010087@mail.ecust.edu.cn (S.Y.); 2 Faculty of Biomedical Engineering, University of New South Wales, Sydney, NSW 2052, Australia; ethan.shen@student.unsw.edu.au

**Keywords:** docetaxel, nanomicelles, allergic reaction

## Abstract

**Background/Objectives**: Docetaxel is a potent anti-cancer agent capable of treating various types of cancer. However, it often induces a range of adverse reactions when used with its standard solubilizer, Tween-80, necessitating allergy prophylaxis with dexamethasone prior to administration. To mitigate the risk of allergic reactions, with nanomicelles garnering significant interest due to their enhanced solubility and thermodynamic stability. **Methods**: In this research, a mPEG-PLA-Lys(Fmoc) micellar carrier with *m* = 45 and *n* = 10 was engineered to encapsulate docetaxel, and its self-assembly into micelles was investigated. Additionally, allergic reaction studies were conducted on animals. **Results**: The findings indicated that the formulation did not cause hemolysis, vascular, or muscle irritation in rabbits, nor did it elicit an allergic response in guinea pigs. **Conclusions**: These results suggest that nanomicelle-encapsulated docetaxel can diminish the allergic reactions associated with docetaxel injections, offering a novel approach to enhance the therapeutic utility of this outstanding anti-cancer drug.

## 1. Introduction

Docetaxel (DTX) is an anthranilic acid anti-cancer compound, prepared semi-synthetically from the extraction of paclitaxel from the European yew tree [1]. DTX can treat various types of cancer, including breast cancer, head and neck cancer, lung cancer, stomach cancer, etc., and is an essential chemotherapy medicine for breast cancer patients after surgery [2]. Its anti-cancer mechanism can be summarized as binding to free tubulin, promoting its assembly into stable microtubules, while inhibiting breakdown [3]. Therefore, it can prevent cancer cell division, leading to cancer cell apoptosis.

The current commercially available DTX injection in clinical practice can bring various issues. DTX itself can cause allergic reactions in patients, such as dyspnea, bronchospasm, urticaria, flushing, rash, hypotension, angioedema, etc. [4]. The cause of the DTX allergic reaction may be mediated by type I reaction immunoglobulin E (IgE) or caused by direct drugs acting on mast cells and basophils, resulting in the release of large amounts of histamine [5,6]. Additionally, due to the poor water solubility of DTX, all DTX that is commercially available uses Tween-80 as a solubilizing agent. However, Tween-80 in clinical application still exhibits some serious adverse reactions, such as hypersensitivity [7], myelosuppression, fluid retention, neurotoxicity, and other adverse reactions [8,9], and it requires dilution to the desired concentration with saline or 5% glucose solution before using DTX.

To address the side effect caused by DTX itself and the solubilizer Tween-80, the current solution is to use dexamethasone as preclinical medication to reduce the risk of allergic reactions in tumor patients receiving DTX. Injection of dexamethasone 30 min before medication can prevent emesis and nausea caused by DTX [10,11]. However, there are also many side effects associated with the use of dexamethasone. Dexamethasone can induce diabetes, hypertension, and hyperlipidemia, severely affecting the cardiovascular function of patients and increasing the mortality risk of cardiovascular incidences [12,13]. Additionally, one of the most serious adverse reactions caused by dexamethasone is inducing infection, especially in patients receiving breast-conserving surgery and breast reconstruction, which may increase the risk of infection and affect early wound healing [14].

To eliminate the allergic reactions caused by DTX, researchers developed different drug delivery systems, such as nanoparticles (NPs) [15,16,17], solid lipid nanoparticles [18,19,20], liposomes [21,22,23], nanomicelles [24,25,26], and other drug delivery systems. Among them, nanomicelles have high solubility (drug loading) for low-water-soluble drugs. Nanomicelles act as direct drug carriers; the critical concentration of micelles is significantly lower, and the lifetime is longer (higher thermodynamic stability) compared with surfactants (low molecular weight) [27,28]. By loading the drug into the nanomicelles, Tween-80 is removed and avoids direct contact of the bloodstream with DTX, as well as waiving the tedious process of pre-allergy before medication. However, it is worth noting that although nanomicelles, especially polyethylene glycol (PEG)-modified micelles, exhibit many advantages in drug delivery, they may also trigger hypersensitivity reactions by activating the complement system, known as complement activation-associated pseudo allergic reactions (CARPA) [29], which may pose new risks to patients. Moreover, Hu [30] prepared biodegradable methoxy poly (ethylene glycol)-poly (L-lactic acid) MPEG-PLA nanomicelles loaded with DTX and Cur for intravenous injection in the study. Taehoon Sim [31] et al. prepared polymer micelles (DTBM) loaded with docetaxel (DTX) using a triblock copolymer polyethylene glycol polylactic acid polyethylene glycol (PEG-PLA-PEG) to comprehensively study its drug application as an anti-cancer nanomedicine.

Leveraging these benefits, a mPEG-PLA-Lys(Fmoc) micellar carrier has been engineered to encapsulate docetaxel (DTX). In this nanocarrier, mPEG-PLA is a Methoxy-Poly(ethylene glycol)-Poly(lactide) (mPEG-PLA) copolymer. Fmoc (fluoroalkyl-meth-oxy-carbonyl) is used to enhance intermolecular interactions between drugs and carriers. The DTX micellar formulation was prepared using the film hydration technique, and subsequent allergic reaction studies were performed on animals. Specifically, the study evaluated the hemolytic, muscle, and vascular irritation effects in New Zealand rabbits, as well as the allergic responses following systemic administration via intravenous injection of DTX micelles in rats. The experimental outcomes with rabbits demonstrated no evidence of hemolysis or coagulation and no vascular or muscle irritation in response to the DTX micelles. Furthermore, intravenous administration of the DTX micelles to surviving guinea pigs did not provoke any allergic reactions.

## 2. Experimental Materials and Experimental Animals

### 2.1. Materials

mPEG-PLA-Lys(Fmoc) micelle carrier has a purity > 99.5%. The preparation method [32] was derived from previous research by our team: ethanol absolute; 0.9% sodium chloride injection, purity > 99.9%; Freund’s adjuvant complete, purity > 99%; Freund’s adjuvant incomplete, purity > 99%; Evans blue, purity > 99%. The reagents above were purchased from Sinopharm Chemical Reagent Co., Ltd. (Shang, China). DTX was purchased from Sanofi S.A. (Paris, France). Bovine Serum Albumin was purchased from Beijing HFK Bio-Technology Co., Ltd. (Beijing, China).

### 2.2. Experimental Apparatus

Electronic balance, DT2000A, Shanghai YIHENG Scientific Instrument Co., Ltd. (Shanghai, China); vernier caliper, Shanghai HuXi Analysis Instrument Factory (Shanghai, China); rotary evaporator, RE-52AA, Shanghai Yarong Biochemical Instrument Factory (Shanghai, China); table top low speed centrifuge, LD5-10B, Beijing Jingli Centrifuge Co., Ltd. (Beijing, China).; electrothermal thermostatic water bath, SSW-420-2S, Shanghai Boxun Medical Biological Instrument Corp. (Shanghai, China); two-channel syringe pump, WZS-50F6, Zhejiang Smiths Medical Science Instrument Co., Ltd. (Zhejiang, China); pathological tissue microtome, RM2235; pathological tissue embedding machine, EG1150; pathological tissue dehydrator, ASP300S; pathological tissue water bath, HI1210; pathological tissue autostainer, ST5010; pathological tissue slide dryer, HI1220; pathological film sealing machine, CV5030; microscope, DM3000. The instruments above were purchased from Leica, (Wetzlae, Hesse, Germany). The microscope 80i was purchased from Nikon Corporation (Tokyo, Japan). This study was approved by the Bioethics Committee of East China University of Science and Technology, with ethics code ECUST-2024-032.

### 2.3. Animals

Thirty-two specific pathogen-free (SPF) Sprague Dawley (SD) rats, weighing between 151 and 175 g, with an equal number of males and females; eight conventional New Zealand rabbits, weighing between 2.07 and 2.25 kg, with an equal number of males and females; twenty-four healthy conventional guinea pigs, all with white fur, weighing between 302 and 337 g, with an equal number of males and females. All animals were purchased from Shanghai Sippe-Bk Animal Co., Ltd. (Shanghai, China). These animals were provided with municipal drinking water, which was treated by a LAWS-2000 (Shanghai, China) experimental animal reverse osmosis water purifier and then placed into sterilized drinking water bottles for consumption. All animal experiments are strictly conducted in accordance with the guiding principles issued by the State Food and Drug Administration [33,34]. This research has been approved by the Bioethics Committee of East China University of Science and Technology, with ethics code: ECUST-2024-032.

### 2.4. Muscle Stimulation Test

Eight healthy New Zealand rabbits with unimpaired limbs and muscles, post-quarantine clearance, were randomly assigned to two groups of four, with an equal number of males and females in each. In the treatment group, the quadriceps muscles of both the left and right legs were injected with docetaxel micelles (1 mL containing 0.74 mg per side, once daily), while the control group received an injection of 0.9% sodium chloride solution (1 mL per side, once daily). The injection sites were monitored for signs of erythema and edema at pre-injection, 24 h, and 48 h post-injection. Systemic symptoms in the rabbits, such as ruffled fur, lethargy, anorexia, and mobility issues, were also observed. The animals were euthanized under anesthesia 48 h after the administration, and the changes in the quadriceps muscle tissue at the injection site were examined through dissection. Subsequently, histopathological assessments were conducted to evaluate any tissue alterations.

### 2.5. Hemolysis Test

Rabbit blood was collected from the muscle stimulation test from rabbits in the 0.9% sodium chloride injection control group. Using the macroscopic observation method of the drug hemolysis test, we observed DTX micelle tubes No. 1–5 for injection within 3 h at 37 °C. After 3 h, the tubes were centrifuged and the results were analyzed.

### 2.6. Active Allergy Test

Twenty-four conventional white guinea pigs, evenly split by gender, were randomly allocated into four groups. Each group received an intraperitoneal injection according to the following dosing regimen: high-dose DTX micelles at 1 mg/kg, low-dose DTX micelles at 0.5 mg/kg, bovine serum albumin at 60 mg/kg, and 0.9% sodium chloride injection at 2 mL/kg for sensitization, with injections administered every two days for a total of three times. Following the sensitization period, a single rapid intravenous injection was administered 10–14 days after the last sensitization to each group as follows: high-dose DTX micelles at 2 mg/kg, low-dose DTX micelles at 1 mg/kg, bovine serum albumin at 120 mg/kg, and 0.9% sodium chloride injection at 4 mL/kg for stimulation. Throughout the sensitization phase, the animals were monitored daily for any signs of adverse effects, and their weights were recorded on the first and last days of sensitization and stimulation. Immediately to 30 min post intravenous stimulation, the animals were closely observed, and any occurrences of restlessness, piloerection, shivering, rhinorrhea, sneezing, coughing, rapid breathing, urination, defecation, tearing, dyspnea, wheezing, purpura, abnormal gait, jumping, gasping, spasms, spinning, Cheyne-Stokes respiration, or death were noted. The onset and resolution times of these symptoms were meticulously documented.

### 2.7. Passive Allergy Test

Twenty-four SD rats, evenly distributed by gender (half male and half female), were selected and divided into six groups. Following a 48 h period of passive sensitization via intradermal injection of 0.1 mL of antiserum, each group received an intravenous injection of either high-dose DTX micelles at 2 mg/kg, low-dose DTX micelles at 1 mg/kg, bovine serum albumin at 100 mg/kg, or 0.9% sodium chloride injection at 10 mL/kg, along with 1% Evans Blue solution at 1 mL per rat for excitation purposes. Thirty minutes post-stimulation, the animals in each group were euthanized. The dorsal skin was then shaved, and the dimensions of the Evans Blue-stained area within the skin’s inner layer were measured to assess the allergic response.

### 2.8. Vascular Irritation Test

Eight New Zealand rabbits, with no ear injuries and cleared from quarantine, were randomly assigned to two groups, each consisting of four rabbits with an equal gender distribution. The groups were the DTX micelle injection group, receiving a dosage of 7.4 mg (10 mL) per kilogram per day, and the 0.9% sodium chloride injection control group, receiving 10 mL per kilogram per day. Each rabbit in the study had the left ear administered with the respective solution. Detailed observations and recordings were made of the stimulation responses in the veins (blood vessels), surrounding subcutaneous tissue, and ear epidermis at the injection site, both before the administration and at 48 to 96 h post-administration.

## 3. Construction of Self-Loading Micelles

### Different Construction Methods of Micelles

DTX and mPEG-PLA-Lys (Fmoc) excipients with different mass ratios (e.g., 1:5, 1:10, etc.) were accurately weighed and dissolved in a container containing ethanol–water (volume ratio 1:3 to 1:5) solvent mixture. When the quality ratio was 1:20, a mixture of 10 mg of docetaxel and 190 mg of mPEG-PLA-Lys(Fmoc) was dissolved in 5 mL of ethyl acetate at 40 °C. The solvent was then gradually evaporated under vacuum to form a thin film, which was subsequently hydrated with 5 mL of ultrapure water. The resulting micellar dispersion was filtered through a 0.22 μm PVDF filter to eliminate any insoluble impurities and large particles. Using the dialysis method, the solution was transferred into an 8000–10,000 Da dialysis bag, dialyzed in distilled water for 24–48 h while stirring and changing the water. The thin-film dispersion method was to dissolve drugs and excipients in a small amount of volatile solvents such as chloroform, rotate and evaporate into a film, add distilled water, and disperse with water bath ultrasound for 10–30 min (200–400 W). The ultrasound method involved sonication of the solution mixture at 20–40 kHz and 300–500 W for 15–30 min (temperature controlled to 0–10 °C in an ice bath). Then, DLS was applied to measure the particle size and electric potential and conduct a comprehensive consideration of various factors such as particle size, distribution, and potential stability to select the optimal construction scheme with a particle size of 10–30 nm, an absolute potential value greater than 20 mV, and a narrow particle size distribution, in order to provide high-quality micelle samples for subsequent research.

## 4. Experimental Results

### 4.1. Analysis of Self-Loading Micelle Construction Result

**Figure 1:**
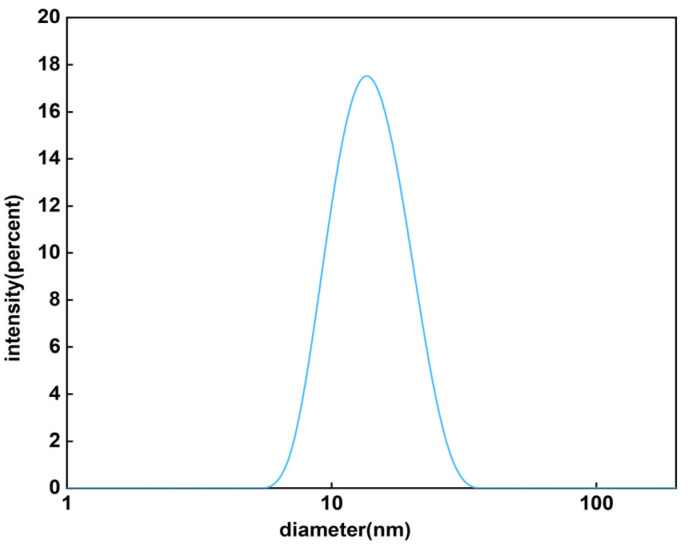
Particle size distribution of DTX micelles.

Referring to a previous study [30], a conclusion was made that when the mPEG-PDLLA-Lys (Fmoc) carrier was at *m* = 45 and *n* = 10 (where m is the degree of polymerization of PEG segments and n is the degree of polymerization of PLA segments), the particle size distribution of micelles was relatively concentrated with an average particle size between 20 and 30 nm. This size range was beneficial to the circulation and penetration of micelles in the body, as shown in Figure 1. The critical micelle concentration measured by the fluorescence probe method is between 0.98 and 1.5 μg/mL. According to Table 1, the micelles prepared by dialysis had a wide range of particle size distribution under different ratios of drugs and excipients; the micelle particle size obtained by the thin-film dispersion method had a relatively centralized distribution, and the micelle particle size trend prepared by the ultrasound method was similar to the thin-film dispersion method, but the overall particle size was slightly larger.

The micelles prepared by the dialysis method have a wide particle size distribution range under different drug to excipient ratios. When the ratio of drug to mPEG PDLLA Lys (Fmoc) is 1:5, the average particle size is about 40 ± 10 nm. When the ratio is 1:10, the average particle size decreases to 25 ± 8 nm. At a ratio of 20, the average particle size further decreased to 18 ± 5 nm. The particle size of micelles obtained by the thin-film dispersion method is relatively concentrated, with a particle size of about 30 ± 6 nm when the ratio of drug to excipient is 1:5. At a ratio of 10, it is 20 ± 4 nm. At a ratio of 20, it is 15 ± 3 nm. The particle size change trend of micelles prepared by the ultrasonic method is similar to that of the thin-film dispersion method, but the overall particle size is slightly larger, with a particle size of 35 ± 8 nm at a ratio of 1:5. At a ratio of 10, it is 22 ± 5 nm. At a ratio of 20, it is 16 ± 4 nm. Overall, when the ratio of drug to mPEG PDLLA Lys (Fmoc) is 1:20, the particle size of the micelles prepared by the three methods is ideal, and the particle size distribution of the thin-film dispersion method is narrower, and the stability may be higher.

The micelles prepared by the analytical method have a drug loading capacity of about 10% and an encapsulation efficiency of 60% when the drug to excipient ratio is 1:5. At a ratio of 10, the drug loading is 8%, and the encapsulation efficiency is 75%. At a ratio of 20, the drug loading is 5%, and the encapsulation efficiency is 90%. The micelles prepared by the thin-film dispersion method have a drug loading of 12% and an encapsulation efficiency of 65% at a ratio of 1:5. At a ratio of 10, the drug loading is 9%, and the encapsulation efficiency is 80%. At a ratio of 20, the drug loading is 6%, and the encapsulation efficiency is 95%. The micelles prepared by the ultrasonic method have a drug loading of 11% and an encapsulation efficiency of 62% at a ratio of 1:5. At a ratio of 10, the drug loading is 8.5%, and the encapsulation efficiency is 78%. At a ratio of 20, the drug loading is 5.5%, and the encapsulation efficiency is 92%. The results showed that as the ratio of drug to excipient decreased, the encapsulation efficiency increased, and the drug loading decreased. The film dispersion method had the highest encapsulation efficiency and relatively balanced drug loading at a ratio of 1:20.

In summary, when the ratio of drug to mPEG-PDLLA-Lys (Fmoc) is 1:20, the micelle particle size prepared by the three methods is ideal, and the particle size distribution of the thin-film dispersion method is narrower, and the stability may be higher. With the decreasing ratio of drug to excipients, the absolute value of micelle electric potential gradually increased, and the stability was enhanced; in the film dispersion method, the absolute value of micelle reached the highest electric potential at the ratio of 1:20 with relatively high stability. However, with a decreasing ratio of drug to excipients, the encapsulation efficiency increased, and the drug loading decreased. The encapsulation efficiency was up to 95% at the ratio of 1:20 in the thin-film dispersion method with relatively balanced drug loading. After a comprehensive analysis of the data under different preparation methods and conditions, a determination was made that when *m* = 45, *n* = 10 (the corresponding ratio of the drug to mPEG-PDLL-Lys (Fmoc) was 1:20), the thin-film dispersion method was selected as the best construction scheme.

### 4.2. Muscle Irritation Experiment Result

**Figure 2:**
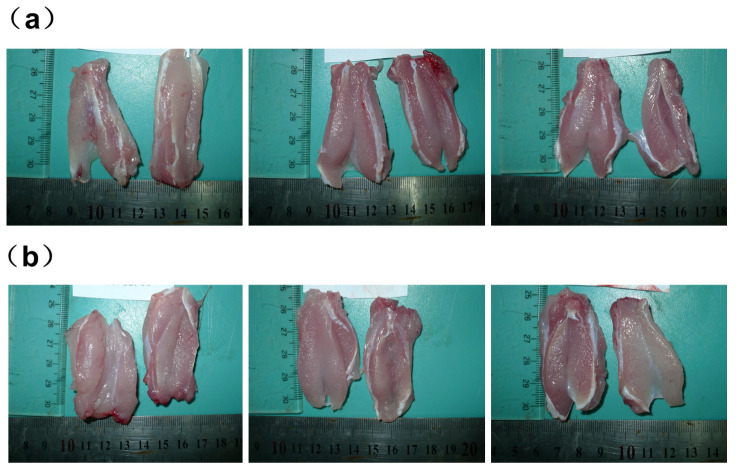
(**a**) Rabbits quadriceps muscles on two sides in the 0.9% sodium chloride injection control group; (**b**) rabbits quadriceps muscles on two sides in the DTX micelle injection group.

**Figure 3:**
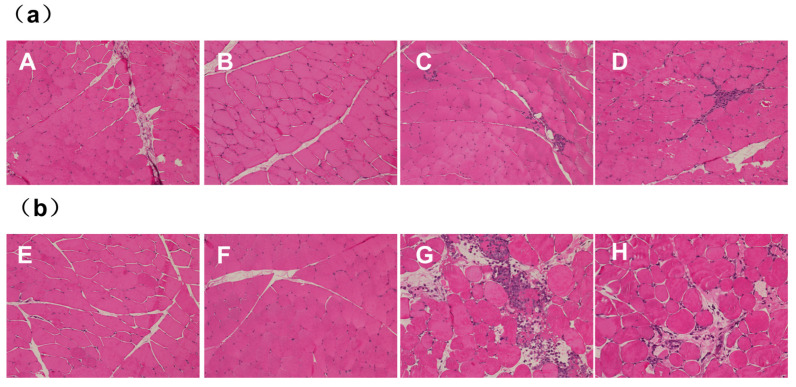
(**a**) Histologic section of rabbits’ quadriceps muscles on two sides in the 0.9% sodium chloride injection control group; (**b**) histologic section of rabbits’ quadriceps muscles on two sides in the DTX micelle injection group.

The quadriceps muscles of both the left and right legs of the rabbits were injected with either DTX micelles at a dosage of 0.74 mg (1 mL) per side or an equal volume of 0.9% sodium chloride injection (1 mL per side). Autopsies were conducted 48 h after the cessation of drug administration. Gross anatomical observations revealed that the quadriceps muscles in both the control group receiving 0.9% sodium chloride injection (Figure 2a) and the DTX micelle group (Figure 2b) appeared ruddy, uniform, shiny, and elastic to the touch, with no signs of congestion, swelling, or necrosis. Histological examination indicated that only one rabbit’s quadriceps muscle exhibited minimal inflammatory infiltration, primarily consisting of a small number of monocytes in the microfocal interstitium, without any muscle fibrosis or necrosis. The histological examination of the other three rabbits showed no abnormalities, as shown in Figure 3b. Similar findings were observed in two rabbits from the control group that received 0.9% sodium chloride injection, as shown in Figure 3a.

The injection of DTX micelles and 0.9% sodium chloride into the quadriceps muscle of rabbits primarily induced interstitial inflammatory infiltrates. However, these lesions were localized and did not include muscle irritant lesions such as fibrosis or necrosis. A comprehensive evaluation concluded that these local lesions were due to the mechanical irritation of the injection rather than the drug’s muscle irritancy.

The above results indicate that under the dosage conditions of this experiment and the muscle stimulation test response grading standard, intramuscularly administered DTX micelles did not exhibit any stimulating effects on the quadriceps muscles of the test rabbits.

### 4.3. Hemolysis Test Results

**Figure 4:**
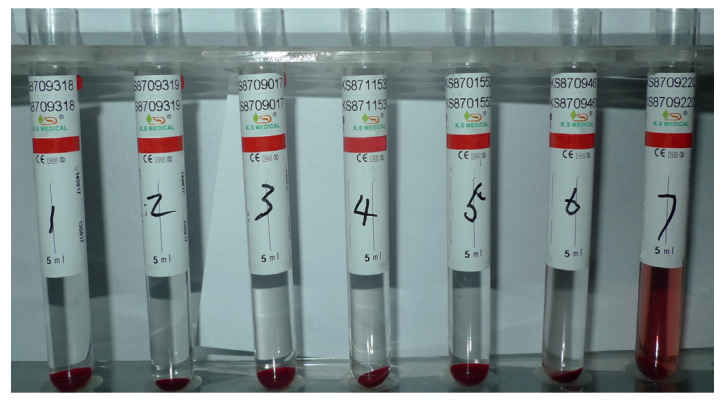
Results of DTX micelle injection hemolysis test.

No observation of hemolysis and agglutination in tubes No. 1–5 of DTX micelle injection at 37 °C within 3 h at each observation time (Figure 4). Centrifugation after 3 h of 37 °C water bath showed that the upper liquid of tubes No. 1–5 was clear and colorless, and the red blood cells condensed to the bottom of the tubes, basically consistent with the 0.9% sodium chloride negative control tube (tube No. 6). Hemolysis occurred in the distilled water positive control tube (tube No. 7) in each observation time. The solution was clear red after centrifugation, with few red blood cells remaining at the bottom of the tube. The above results showed that there was no hemolysis and agglutination reaction in DTX micelle injection under this experimental condition.

### 4.4. Guinea Pig Active Allergy Test Results

Healthy white guinea pigs were randomly assigned to receive intraperitoneal injections of DTX micelles at 1 mg/kg for the high-dose group and 0.5 mg/kg for the low-dose group, bovine serum albumin at 60 mg/kg, and 0.9% sodium chloride at 2 mL/kg for the control group, administered every two days for a total of three sensitization cycles. Throughout the sensitization period, as shown in Table 2, guinea pigs in both DTX micelle groups exhibited reduced food intake, progressive weight loss, and changes in fur texture. Specifically, three guinea pigs in the high-dose group died on the 5th and 7th days post-final sensitization, and one in the low-dose group died on the 5th day post-final sensitization. The average body weight of the high-dose group at the last sensitization and stimulation, and the low-dose group at stimulation, was significantly lower than that of the 0.9% sodium chloride control group at the respective times (*p* < 0.05, *p* < 0.01). In contrast, the bovine serum albumin and 0.9% sodium chloride groups showed no significant adverse reactions, and their body weights increased over the test period, with no statistical difference in average body weight at the first sensitization, last sensitization, and stimulation compared to the 0.9% sodium chloride group (*p* > 0.05).

On day 11 following the final sensitization, the surviving guinea pigs were challenged with a single intravenous injection of the respective test substances at double the sensitization dose. The high-dose and low-dose DTX micelle groups showed no significant adverse reactions, similar to the 0.9% sodium chloride control group, with an allergic reaction and mortality rate of 0%. However, the bovine serum albumin positive control group displayed pronounced allergic symptoms, including restlessness, piloerection, shivering, rhinorrhea, coughing, rapid breathing, urination, lacrimation, dyspnea, abnormal gait, spasms, and death. The median death time was about 5 min after injection. The reaction occurrence and mortality rates in this group were 100%.

Under the experimental conditions and dosages used, DTX micelle injections did not provoke allergic reactions in the surviving guinea pigs. The observed decreased food intake, weight loss, and fur changes in the high-dose and low-dose DTX micelle groups, along with the deaths, were attributed to the inherent toxicity of the drug.

### 4.5. Guinea Pig Passive Allergy Test Results

Under the experimental conditions and dosages used, DTX micelle injections did not provoke allergic reactions in the surviving guinea pigs. The observed decreased food intake, weight loss, and fur changes in the high-dose and low-dose DTX micelle groups, along with the deaths, were attributed to the inherent toxicity of the drug.

**Figure 5:**
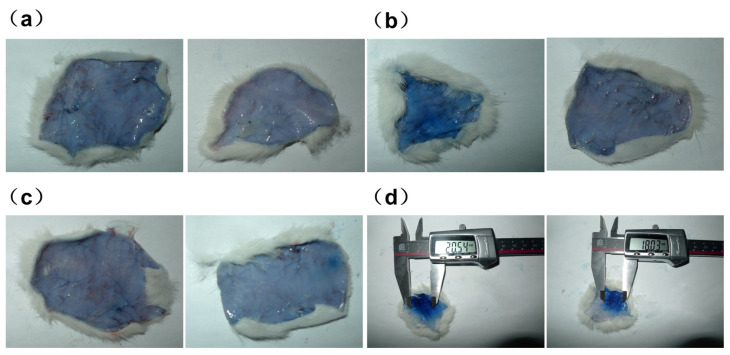
(**a**) Rat skin in high-dose DTX micelle group; (**b**) rat skin in low-dose DTX micelle group; (**c**) rat skin in 0.9% sodium chloride negative control group; (**d**) rat skin in bovine serum albumin positive control group.

According to the anatomical results shown in Figure 5a,b, no blue spots in the inner skin of the rats in the 1 mg/kg dose DTX micelles group and 0.9% sodium chloride negative control group in Figure 5c are observed. In the bovine serum albumin positive control group, as shown in Figure 5d, there were obvious blue spots (diameter greater than 5 mm) in the inner skin of the rats’ backs, with the blue spot reaction being positive. The above results concluded that DTX micelle injection had no passive skin allergic reaction in sensitized rats under this experimental dose.

### 4.6. Vascular Stimulation Test Results

A single, slow intravenous injection of DTX micelles at a dosage of 7.4 mg (10 mL)/kg and an equivalent volume of 0.9% sodium chloride at 10 mL/kg was administered into the auricular vein of rabbits. Throughout the experimental period, no local reactions such as congestion, swelling, or necrosis were observed at any of the monitoring points. Autopsies were conducted 96 h after the cessation of the medication.

**Figure 6:**
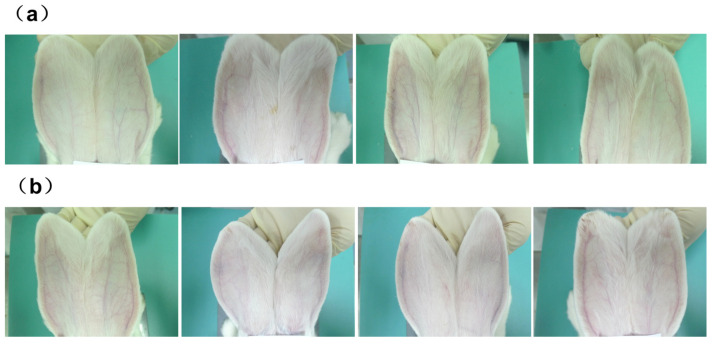
(**a**) Rabbit ears in DTX micelle group; (**b**) rabbit ears in 0.9% sodium chloride control group.

**Figure 7:**
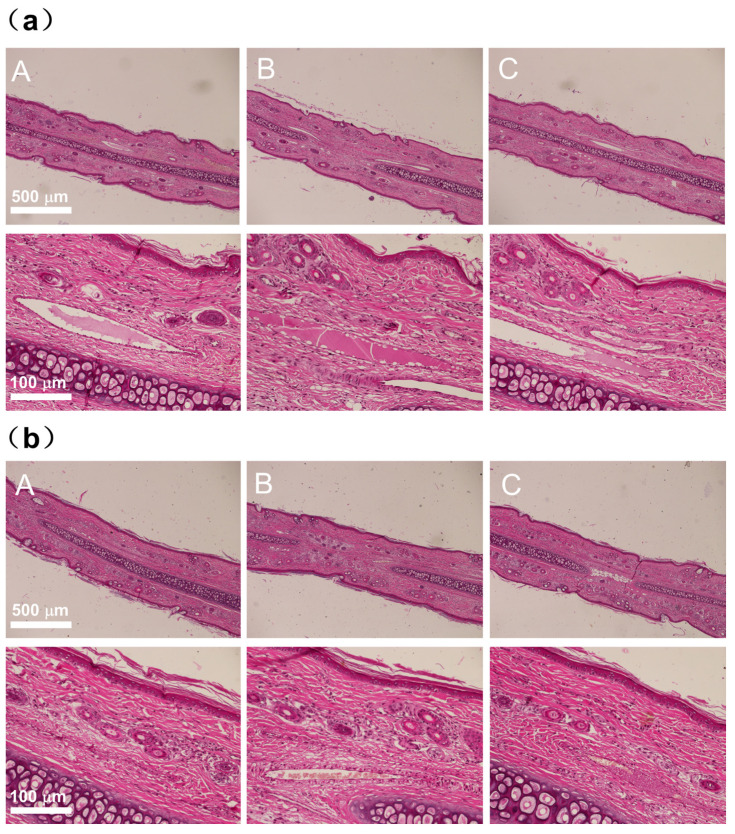
(**a**) Ear tissue and corresponding local magnification of A, B, and C segments on the injection side of the 0.9% sodium chloride control group; (**b**) ear tissue and local magnification of A, B, and C segments on the injection side of the DTX micelle group.

Gross anatomical observations, as depicted in Figure 6, revealed no signs of irritant lesions like congestion, swelling, or necrosis in the ears of rabbits from both the DTX micelle group and the 0.9% sodium chloride control group, with injections made on the left side and no injections on the right side. Histopathological examination indicated that the ear tissue structures of segments A, B, and C in the 0.9% sodium chloride control group (Figure 7a) and the DTX micelle group (Figure 7b) were normal, with no evidence of thrombus, embolism, interstitial bleeding, inflammatory infiltration, or necrosis.

Consequently, under the dosage used in this experiment, the intravenous administration of DTX micelles through the auricular vein did not cause any irritation to the rabbit’s blood vessels.

### 4.7. Characteristics of Nanomicelles

Nanomicelles usually have a small particle size, generally between 10 and 100 nm. This small particle size allows micelles to be better dispersed when circulating in the body, reducing physical stimulation of vascular endothelial cells, thereby reducing the potential for vascular stimulation responses. At the same time, the surface of nanomicelles usually carries a certain charge. For example, the zeta potential of docetaxel micelles in this study is −35 ± 6 mV. This charge can increase the stability of micelles, prevent their aggregation in vivo, further reduce the mechanical damage to blood vessels and muscle tissues, and help avoid hemolysis and muscle stimulation reactions. Moreover, nanomicelles have certain tissue distribution specificity and targeting, which can deliver drugs to target tissues more effectively and reduce the distribution of drugs in non-target tissues. For blood vessels and muscle tissue, reduced drug concentrations mean reduced drug stimulation, resulting in reduced hemolysis and stimulation responses.

In the preparation of nanomicelles, the immunogenicity of drugs can be reduced by selecting appropriate carrier materials and optimizing the preparation process. In addition, impurities or excipients that may cause allergic reactions are not introduced in the preparation process, so that the finally prepared docetaxel micelles are not easy to be recognized as foreign antigens by the immune system of the body, thereby reducing the possibility of triggering allergic reactions. The distribution and metabolic properties of docetaxel micelles in vivo also contribute to reducing the occurrence of allergic reactions. Its high concentration distribution in target tissues allows the drug to exert therapeutic effects more effectively, reducing ineffective distribution and potential immune stimulation in non-target tissues. In addition, the metabolites of micelles are relatively mild, which is not easy to trigger an immune response of the body, further ensuring the safety of micelles in guinea pigs without an allergic reaction.

In conclusion, the particle size, surface characteristics, targeting, and composition of nanomicelles combined with docetaxel micelles did not cause hemolysis and vascular or muscle stimulation in rabbits or allergic reactions in guinea pigs.

## 5. Conclusions

This study focused on the design and investigation of the interaction between docetaxel (DTX) and mPEG-PLA-Lys(Fmoc) copolymer nanomicelles in an aqueous environment. Simulation outcomes indicated that the optimal micellar configuration was achieved with *m* = 45 and *n* = 10, characterized by high stability, desirable particle size and shape, excellent drug loading capacity, and a favorable drug release profile. Guided by these findings, a DTX-loaded micelle formulation was developed and subjected to animal testing. The results demonstrated that this preparation elicited no hemolytic, vascular, or muscular irritation in rabbits and no allergic reactions in guinea pigs. These observations suggest that the DTX micelles, as a novel drug delivery system, can substantially decrease the allergic reactions associated with DTX and its solubilizer Tween-80, thereby enhancing the drug’s anti-tumor efficacy and reducing its toxic side effects. In the future, we will further investigate whether PEG nanomicelles may be involved in complement activation.

## Figures and Tables

**Table 1:** Construction of self-loading micelles and analysis of optimization results.

Preparation Method	Drug to Excipients Ratio	Particle Size (nm)	Electric Potential (mV)	Drug Loading Capacity (%)	Encapsulation Efficiency (%)
Dialysis Method	1:5	40 ± 10	−15 ± 3	10 ± 2	60 ± 3
	1:10	25 ± 8	−22 ± 4	8 ± 1	75 ± 4
	1:20	18 ± 5	−30 ± 5	5 ± 2	90 ± 6
Thin-Film Dispersion Method	1:5	30 ± 6	−18 ± 4	12 ± 3	65 ± 5
	1:10	20 ± 4	−25 ± 5	9 ± 2.5	80 ± 4
	1:20	15 ± 3	−35 ± 6	6 ± 1	95 ± 3
Ultrasound Method	1:5	35 ± 8	−16 ± 3	11 ± 1.5	62 ± 4
	1:10	22 ± 5	−23 ± 4	8.5 ± 2	78 ± 5
	1:20	16 ± 4	−32 ± 5	5.5 ± 1	92 ± 5

**Table 2:** Results of the systemic active allergy test in guinea pigs with DTX micelle injection.

Group	Dosage	Weight (g, X ± s)			Death Rate During Sensitization (%)	Stimulation Response Condition		
		First Sensitization	Last Sensitization	Stimulation		Reaction Occurrence Rate (%)	Death Rate (%)	Conclusion
DTX High Dosage	Sensi.: 1 mg/kg × 3 times Stimu.: 2 mg/kg × 1 time	326 ± 11	325 ± 14 ^*^	310 ± 15 ^**^	50	0	0	Negative
DTX Low Dosage	Sensi.: 0.5 mg/kg × 3 times Stimu.: 1 mg/kg × 1 time	324 ± 14	331 ± 24	348 ± 28 ^**^	16.7	0	0	Negative
Bovine Serum Protein	Sensi.: 60 mg/kg × 3 times Stimu.: 120 mg/kg × 1 time	331 ± 15	347 ± 11	385 ± 10	0	100	100	Positive
0.9% NaCl	Sensi.: 2 mL/kg × 3 times Stimu.: 4 mL/kg × 1 time	330 ± 8	344 ± 10	392 ± 14	0	0	0	Negative

* *p* < 0.05, ** *p* < 0.01.

## Data Availability

The raw data supporting the conclusions of this article will be made available by the authors on request.
